# Utilisation and prognostic impact of cathlab investigation prior to intensive care admission for patients following out-of-hospital cardiac arrest

**DOI:** 10.1186/cc14502

**Published:** 2015-03-16

**Authors:** L Eveson, S Shrestha, V Achan, M Davies, M Peck

**Affiliations:** 1 Frimley Park Hospital, Frimley, UK

## Introduction

Our 700-bed hospital has a 24-7 cathlab service that routinely investigates patients with indications prior to ICU admission following out-of-hospital cardiac arrest (OOHCA). Our aim was to compare survivors and nonsurvivors and evaluate utilisation and prognostic impact of angiography and primary percutaneous coronary intervention (PPCI) in this patient group.

## Methods

A retrospective analysis using Trust electronic databases (Symphony, WardWatcher, PICIS, PRISM) of all OOHCA patients admitted to our ICU over 3 consecutive years between 1 November 2011 and 31 October 2014.

## Results

A total of 351 patients presented to our emergency department (ED) following OOHCA in this period, and of these 50% died in the ED, 37% were admitted to the ICU and 13% were admitted elsewhere. Of the 129 patients admitted to the ICU, median age was 66 (range 18 to 93), 71% were male, 68% had a shockable presenting rhythm, median ICU LOS was 3.75 (range 1 to 34 days) and ICU and hospital mortalities were 50% and 60% respectively. Eighty-nine percent (*n *= 48) of OOHCA survivors admitted to the ICU had a shockable rhythm compared with 55% (*n *= 41) of nonsurvivors. Eighty-three percent (*n *= 45) of survivors admitted to the ICU went to the cardiac cathlab before ICU admission compared with 53% (*n *= 39) of nonsurvivors. Forty-three percent of survivors had PPCI compared with 26% of nonsurvivors. Eighty-one percent (*n *= 44) of survivors received therapeutic hypothermia compared with 62% (*n *= 48) of nonsurvivors.

## Conclusion

Over 3 consecutive years our annual case mix, ICU and hospital mortalities for OOHCA patients admitted to the ICU have remained stable, while our annual pre-ICU cathlab and PPCI utilisation have risen consistently in both survivors and nonsurvivors. ICU survivors were more likely to have had a shockable rhythm, been to the cathlab, and received PPCI and TH, but all may simply reflect selection bias. Any benefit these conferred to cardiac patients may have been off set by our liberal ICU admission policy to OOHCAs with nonshockable rhythms. Access to 24-7 PPCI in this group may determine survival and we suggest that OOHCA patients should be taken directly to regional heart attack centres.

